# Kawasaki disease triggered by EBV virus in a child with Familial Mediterranean Fever

**DOI:** 10.1186/s13052-019-0717-8

**Published:** 2019-10-18

**Authors:** Maria Cristina Maggio, Carmelo Fabiano, Giovanni Corsello

**Affiliations:** 1 Department of Health Promotion Sciences Maternal and Infantile Care, Internal Medicine and Medical Specialities “G. D’Alessandro”, University of Palermo, Palermo, Italy; 2 U.O. Laboratorio di Genetica Molecolare, Ematologia e Malattie rare del sangue e degli organi emopoietici, Azienda Ospedaliera Ospedali Riuniti “Villa Sofia-Cervello” - Presidio Cervello, Palermo, Italy

**Keywords:** Familial Mediterranean fever, Kawasaki disease, Epstein Barr virus

## Abstract

**Background:**

Familial Mediterranean Fever is a monogenic autoinflammatory disease, secondary to mutation of MEFV gene, and typically expressed with recurrent attacks of fever, serositis, rash, aphthous changes in lips and/or oral mucosa.

Kawasaki Disease, an acute systemic vasculitis with persistent fever (5 or more days), rash, stomatitis, conjunctivitis, lymphadenopathy, changes in extremities, is currently considered a multifactorial autoinflammatory disease.

An infection, as Epstein Barr virus, can be the trigger of Kawasaki Disease.

**Case presentation:**

We describe the clinical case of a 3-year-old boy with Kawasaki disease. Successfully treated with intravenous immune globulin, acetyl salicylate acid, he late developed anaemia and thrombocytopenia. The Epstein-Barr virus infection has been demonstrated and he showed a resolution of the clinical manifestations of Kawasaki disease with the persistence of coronaritis, without aneurisms.

However, for the personal and familial history of monthly recurrent attacks of fever, pharyngitis, abdominal pain, the genetic study of MEFV was performed and demonstrated 3 heterozygous mutations of MEFV (E148Q, P369S, R408Q).

**Conclusions:**

Mutations of MEFV can contribute to increase inflammatory expression in other diseases, as Kawasaki disease.

## Background

Familial Mediterranean Fever (FMF) is a monogenic autoinflammatory disease, secondary to mutations of MEFV gene, typically expressed with recurrent attacks of fever, serositis, rash, aphthous changes in lips and/or oral mucosa [1, 2]. FMF is caused by dysregulation of the inflammasome, a complex intracellular multiprotein structure, commanding the overproduction of interleukin-1(IL-1). The attacks of FMF can recognize a trigger in infections, stress, menses.

Kawasaki Disease (KD), an acute systemic vasculitis with persistent fever (5 or more days), rash, stomatitis, conjunctivitis, lymphadenopathy, changes in extremities (oedema and/or rash of souls and palms), is currently considered a multifactorial autoinflammatory disease [3].

Although the aetiological mechanism of KD remains unknown, recent studies support the hypothesis that a dysregulation of the immune response to infectious triggers is a possible contribution to KD pathogenesis.

Furthermore, superantigens are proteins, which can activate T cells in the peripheral blood, inducing these cells to form a bridge between the T-cell receptor and the major histocompatibility complex class II of antigen presenting cells in the absence of any antigenic peptide presentation. This outcomes in the overproduction of pro-inflammatory cytokines.

Based on serological and polymerase chain reaction (PCR) analyses of clinical specimens, at least 14 species of virus have been reported to be associated with KD, as Parvovirus, Epstein-Barr virus (EBV), Adenovirus, with a different geographic distribution of the disease [3]. Patients with KD show risk loci and polymorphisms identified by genome wide association studies. Genetic polymorphisms associated with KD susceptibility and resistance to IVIG therapy are documented in the gene encoding inositol-triphosphate 3-kinase C (ITPKC) [4], in the CASP3 gene [5], in the gene encoding ORAI1, a calcium release-activated calcium channel protein 1, with a possible influence on the immune response to EBV infection [6].

Some studies indicate that KD could be associated with dysregulated innate immune response. Its inadequate regulation rises inflammatory tissue damage and increases the inflammatory response.

In a KD mouse model, myocarditis, coronary artery vasculitis and aortitis are induced with an injection of a cell wall extract from *Lactobacillus casei*. The mice manifest increased body temperature, systemic inflammation, associated with elevated levels of IL-1β. In addition, IL-1 related genes are upregulated in KD peripheral blood during acute phase of illness [7]. Studies in the *Lactobacillus casei*-induced mouse model of KD showed that the NLRP3 inflammasome, caspase 1 activation, IL-1β and IL-1α are involved in KD vasculitis.

The polymorphisms of the ITPKC gene were significantly associated with KD in Japan and US [4].

Notably, the ITPKC regulates intracellular Ca^2+^ influx and the mutation of the kinase leads to increased intracellular Ca^2+^, which induces NLRP3 inflammasome activation [8].

Furthermore, some studies evaluated the benefits of IL-1 blockade with anakinra, obtaining a reduction in inflammation.

IL-1Ra pre-treatment significantly reduced KD-induced myocardial inflammation and N-terminal pro B-type natriuretic peptide release, improved ejection fraction and a normalized left ventricular function, *Lactobacillus casei*-induced mouse model of KD. These data support the promising beneficial effects of IL-1Ra therapy in preventing the complications (myocarditis and myocardial dysfunction, coronaritis) in the acute phase of KD [9].

The central role of IL-1 in KD pathogenesis, supports the consideration that KD can be considered as a multifactorial autoinflammatory disease.

Many clinical features of KD are similar to those described in systemic autoinflammatory diseases (SAID), including systemic-onset juvenile idiopathic arthritis (SoJIA): sudden and seemingly unprovoked onset of fever, skin rash, eye and mouth inflammation, cervical adenitis, serositis, pericarditis. Marked elevation of acute phase reactants, high platelets and neutrophils count, anaemia, hypoalbuminemia are common in KD and in soJIA. Intriguingly, transient coronary abnormalities have also been documented in patients with soJIA [10].

KD patients are treated with intravenous immune globulins (IVIG) (2 g/kg) and acetyl salicylate acid (ASA) (30–50 mg/kg/day); lowered to 3–5 mg/kg/day at when afebrile for 48–72 h. In non-responders to IVIG, in patients with a severe outcome or high risk of coronaritis, a second step treatment is required, with high doses of steroids, anti-IL-1 biologic drugs, with documented safety profile in children [11] and immune suppressive drugs [12, 13].

Recent studies support the critical role for IL-1 β in the pathogenesis of SAID and KD. Furthermore, IL-1 polymorphisms could be associated with response or resistance to IVIG. Interestingly elevated transcripts have been shown in IVIG-resistant KD patients, those carrying the highest risk for coronary aneurysms [14, 15].

## Case presentation

We report on a 3-year-old boy with fever persisting for 6 days, non-secretive conjunctivitis, lymphadenitis of the neck, generalized, fixed rash, more expressed in the perineal Xregion. He presented hepatosplenomegaly, gallbladder hydrops, neutrophil leucocytosis, hyponatraemia (128 mEq/l), hypoalbuminemia, significant increase of C-reactive protein (CRP) 13 mg/dl; erythrocyte sedimentation rate (ESR): 57; D-dimer. 836 ng/ml; AST, ALT, gamma-GT (1.5 x n.v.). Abdominal ultrasound documented hepatosplenomegaly with an aspect of “starry sky liver”.

Incomplete KD was the diagnosis of the child and he received IVIG (2 g/kg) and ASA (50 mg/kg/day). Fever resolved 12 h after the IVIG infusion. However, he showed anaemia (8 g/dl), thrombocytopenia (78.000/mm^3^), with a reduction and normalization of CRP and leukocytes number.

Echocardiographic study evidenced coronaritis without aneurisms.

Serology for Epstein-Barr Virus (EBV) was detected before IVIG infusion (for fever with cholestatic hepatitis and the skin rash [16]), and showed a positive IgM EBV-VCA (78,3 UI/ml), confirmed by PCR for EBV.

13 days after the onset of the fever he showed thrombocytosis (687.000/mm3) and, the day after, he presented perineal and subungual desquamation.

Moreover, familial anamnesis reported recurrent episodes of fever with pharyngitis and undefined nephropathy in the grandmother; recurrent episodes of fever, pharyngitis, abdominal pain every month in the child and in the father.

The genetic study for FMF, TNF receptor-associated periodic syndrome (TRAPS), Mevalonate kinase deficiency (MVK) documented the presence of the MEFV gene heterozygous mutations E148Q (exon 2), P369S, R408Q (exon 3) in the child, in the sibling, in the 6-years-old brother and in the father. All the subjects studied showed increased levels of serum amyloid A (SAA) (> 5–10 x n.v.) far away from recurrent attacks.

## Discussion and conclusions

The conjunction of genetic data with the demonstrated crucial role of IL-1 signature in the experimental mouse model of KD, as well as the upregulation of IL-1 pathway in KD patients, strongly supports the hypothesis that IL-1 pathway plays a key role in KD vasculitis and coronary arteritis.

The association of FMF and KD has been reported in some studies. Clinical and immunological relationships between KD and FMF further support the hypothesis that KD must be considered as an autoinflammatory disease. New insights regarding the pathogenesis of KD revealed the key role of IL-1 signalling in the pathogenesis of KD and of other vasculitis. MEFV mutations are associated with increased risk of vasculitis, as Henoch-Schöenlein purpura and KD. However, MEFV mutations are underdiagnosed in some geographical areas as in Sicily, were KD has a relative high incidence of coronaritis [17].

A study on 138 children affected by KD did not show an increased risk of KD and/or associated coronaritis among the patients with MEFV mutations, as E148Q, P369S, R408Q [18].

However, other studies demonstrated that MEFV mutations can be a risk factor of increased inflammatory expression in other diseases, as soJIA, Bechet disease, recurrent pericarditis, systemic vasculitis, with the proposed definition of “pyrin-associated disorders” [19, 20].

In our case, the association of the 3 mutations, rarely described in the same patient, could be the amplifying factor of a meaningful clinical picture, expressed as incomplete KD and triggered by an infection, as possible trigger both in FMF attacks and in KD [21].

In our patient, the resolution of symptoms and biochemical alterations secondary to KD significantly improved after IVIG infusion. However, EBV infection was the cause of late anaemia and thrombocytopenia, trigger of the KD and reason of the peculiar clinical picture.

The critical role for IL-1 β in the pathogenesis of KD create a bridge between KD and SAID, as FMF, explaining the pathogenetic role of MEFV mutations in the described case.

Further studies are necessary to clarify the possible link between a dysregulation of innate immune response to infections, monogenic SAID and KD: the interpretation of KD as a multifactorial autoinflammatory disease contributes to a new treat-to-target approach.

## Data Availability

Materials and data of the patient are included in the medical records of the patient.

## Abbreviations

ASA: Acetyl salicylate acid
CRP: C-reactive protein
EBV: Epstein-Barr virus
ESR: Erythrocyte sedimentation rate
FMF: Familial Mediterranean Fever
IL-1: Interleukin-1
ITPKC: Inositol-trisphosphate 3-kinase C
IVIG: Intravenous immune globulins
KD: Kawasaki Disease
MVK: Mevalonate kinase deficiency
PCR: Polymerase chain reaction
SAA: Serum amyloid A
SAID: Systemic autoinflammatory diseases
SoJIA: Systemic-onset juvenile idiopathic arthritis
TRAPS: TNF receptor-associated periodic syndrome
